# High-temporal-resolution quasideterministic dynamics of granular stick-slip

**DOI:** 10.1038/s41598-021-82581-x

**Published:** 2021-02-03

**Authors:** T. T. T. Nguyen, T. Doanh, A. Le Bot, D. Dalmas

**Affiliations:** 1 Ecole Nationale des Travaux Publics de l’Etat, LGCB, LTDS (UMR 5513), Vaulx en Velin, France; 2 Ecole Centrale de Lyon, LTDS (UMR 5513), Ecully, France

**Keywords:** Rheology, Phase transitions and critical phenomena

## Abstract

We report high-temporal-resolution observations of the spontaneous instability of model granular materials under isotropic and triaxial compression in fully drained conditions during laboratory tests representative of earthquakes. Unlike in natural granular materials, in the model granular materials, during the first stage of the tests, i.e., isotropic compression, a series of local collapses of various amplitudes occurs under random triggering cell pressures. During the second stage, i.e., shearing under triaxial compression, the model granular samples exhibit very large quasiperiodic stick-slip motions at random deviatoric triggering stresses. These motions are responsible for very large stress drops that are described by power laws and are accurate over more than 3 decades in logarithmic space. Then, we identify the quasideterministic nature of these stick-slip events, assuming that they are fully controlled by the cell pressure and solid fraction. Finally, we discuss the potential mechanisms that could explain these intriguing behaviors and the possible links with natural earthquakes.

## Introduction

The search for a method of detecting and preventing earthquakes presents a great challenge and remains an elusive goal for scientists, despite decades of theoretical and experimental studies^1,2^. Model granular materials with spherical particle shapes in thin sheared layers are often used as proxies for fault zones in geophysics, resulting in small quasiperiodic stick-slip motion, contrasting with the irregular nature of earthquakes^3–5^. The dynamics of the stick-slip phenomenon are of particular interest, especially the sudden stress drop and the fast axial strain jump during the slip phase^6,7^, and should be captured in laboratory representatives of earthquakes. The statistical distributions of these dynamics are often explored using the recently proposed mean-field theory, leading to some universal scaling relations^8,9^. Indeed, the main traditional statistical relations of seismicity are even quantitatively reproduced in laboratory shear experiments^10^. However, the precise nature of these stick-slip motions remains unclear, as well as the origin of their physical triggering mechanisms.

In earthquake dynamics, the long-standing question of the deterministic nature of earthquakes is still a matter of debate. Studies on the same seismic datasets can result in a strong determinism in the prediction of earthquake magnitude when based on the first few seconds of earthquake records^11,12^ or a completely diverging and thus nondeterministic perspective^13,14^. Recently, an additional perspective of weak determinism has emerged to distinguish large earthquakes after a common initiation phase of short duration^15,16^, providing an improved and effective basis for earthquake early warning systems.

The discovery of very large stick-slip motions in loose and saturated model granular materials under drained triaxial compression^17^ presents a unique opportunity to elucidate some details of the mechanisms of catastrophic earthquake-like events in the laboratory, especially the fast outburst of pore fluid pressure, which is a distinctive signature of these instabilities. These intriguing experimental observations raise many questions about their origins and interpretation. Indeed, to our knowledge, these dynamic instabilities cannot be theoretically predicted, despite numerous advanced constitutive models for geomaterials^18–24^, including various evolutionary laws of rate-and-state-frictional behavior^3–5^, and the theoretical framework of frictional dynamics^25,26^; even numerically, these dynamic instabilities cannot be predicted, as revealed within an ever-growing body of work on DEM (discrete element model) simulations with various particle shapes^27–34^ and they are difficult to accurately measure experimentally.

In this paper, we provide the first assessment of quasideterministic dynamics of stick-slip motion occurring in slowly sheared triaxial compression experiments^17,35–38^ performed on model granular media. We report the rare vanishing frictional stress during the stick-slip phenomenon. The presence of pore fluid in a fully saturated simple two-phase granular assembly is essential in creating the largest laboratory earthquakes, which result in complete destruction of the granular structure^39^.

With high-temporal-resolution data, we clarify the dynamics of the slip phase and demonstrate the existence of scaling relations in estimating the macroscopic mechanical parameters based on the measurements of vertical top cap acceleration, specifically the short-lived first peak occurring within the first few milliseconds. We explore the role of pore pressure outbursts; while pore pressure outbursts are not a causative mechanism, they lead to a rational explanation of stick-slip events induced by drained triaxial compression with dynamic consolidation under constant deviatoric stress. We consistently identify the uncoupling effects of pressure and the solid fraction and a common slick-slip boundary in the effective stress plane and their influence on granular instability. These observational macroscopic results can be combined with future numerical discrete element studies to identify potential changes inside the granular micro- and mesostructure that may control dynamic slip triggering.

**Figure 1:**
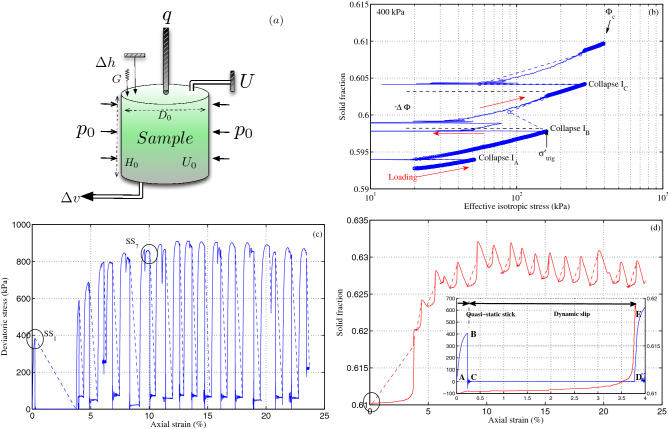
Dynamic instabilities of model granular materials in triaxial compression. (**a**) Sketch of the experimental setup of drained isotropic and triaxial compression on a cylindrical sample inside a triaxial cell under constant radial stress $p_0$. The axial displacement $\Delta h$ and the water volume $\Delta v$ were measured to estimate the global axial strain $\varepsilon _{a}$ and solid fraction $\Phi$. The constant back pressure $U_0$ was applied at the sample bottom, while the measurement of static top pore-water pressure *U* at the sample top allowed us to assess the homogeneity of the effective stress state. One complementary piezoelectric accelerometer *G* measures the vertical acceleration of the sample top cap. (**b**) Collapses in isotropic drained compression from 20 up to 400 kPa. The red arrows denote the direction of loading. The dashed blue lines represent the usual response with a low sampling rate. A semilogarithmic scale is used to emphasize the low range of stress. $\Phi _{c}$ is the uncontrolled solid fraction at the end of this isotropic compression. (**c**) Realistic stress–strain behavior of the stick-slip experiment under compression at a constant confining stress of 400 kPa and a constant axial displacement rate of 0.1 mm/min, showing a large deviatoric stress drop. (**d**) Coupling solid fraction with stick-slip-like behavior. Realistic stress–strain and solid fraction behaviors of the first stick-slip event SS$_1$ in the inset. Only two points, B and D, are accessible using conventional laboratory equipment with a low sampling frequency. (Fig. S4 for additional details).

**Figure 2:**
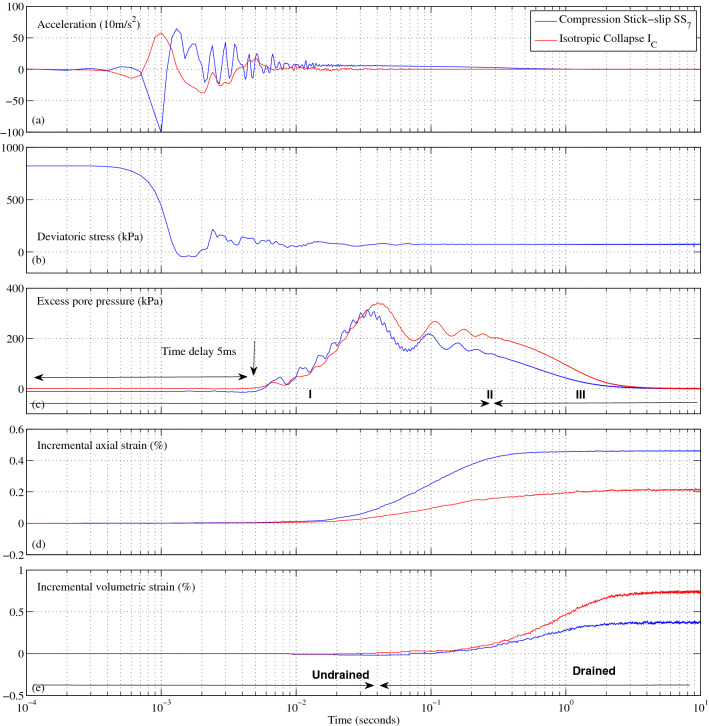
Typical temporal evolution of one representative stick-slip event induced by compression under drained conditions (blue) picked on the deviatoric stress plateau and one isotropic collapse event (red) during the triaxial test at 400 kPa of confining stress. From top to bottom: vertical top cap acceleration, deviatoric stress, excess pore fluid pressure, incremental axial strain and incremental volumetric strain. The excess pore pressure corresponding to the stick-slip event is below the constant cell pressure of 400 kPa, indicating a nonliquefaction event during which the effective stress remains positive. In isotropic compression, the vertical acceleration of the sample top cap is magnified by a factor of 10.

## Experimental setup

We study a short cylindrical sample of small soda-lime glass beads isotropically confined in a triaxial cell (Fig. 1a), which is one of the most popular mechanical systems for investigating the stick-slip phenomenon in granular systems^17,36,40,41^. The loose granular assembly is fully saturated (no air pores), and the test is fully drained (i.e., the fluid is free to escape from the sample if the pore fluid pressure *U* is greater than the prescribed constant back pressure $U_{0}$).

A typical triaxial test in this study comprises two successive loading steps. The first step is isotropic drained compression under a stress-controlled mode with a constant stress rate. Compressed air is used to set a desired final cell pressure $p_0$. Under drained conditions, this isotropic compression generates no macroscopic shear stress and is known as isotropic consolidation in soil mechanics. This simple isotropic loading step is often used to create isotropic granular structures prior to triaxial compression.

Once isotropic compression is completed and if the sample shape is still geometrically stable, the second step consists of drained triaxial compression under a strain-controlled mode with a constant cell pressure and a constant axial strain rate. This second loading step is aimed to increase the deviatoric stress *q*; with increasing axial stress until failure while keeping a constant radial stress, i.e., equal to the initial cell pressuree $p_0$. This vertical compression develops a macroscopic shear stress on an inclined failure plane through the sample.

In these two separate loading steps, the sample is not kinematically constrained in the vertical direction since the vertical loading ram is not physically connected to the sample top cap. Consequently, the sample top cap is free to move vertically (details in the Supplementary Information).

During the two loading steps (isotropic and triaxial compression), a low radial stress rate (approximately 1 kPa/s) for the first step and a low axial strain rate (0.0048%/s) for the second step are required to respect the requirement of the quasistatic shearing regime and that of full drainage by keeping a very low excess pore fluid pressure *U* in the range of a few kilopascals. The static pore-water pressure *U* (Sedeme, MD20) with an unknown resonant frequency, complemented by a dynamic piezoelectric pore pressure sensor (PCB S112A21) with a high resonant frequency (250 kHz) to verify the possible oscillating nature of *U*. The sample axial changes $\Delta h$ are measured by LVDT (Linear Variable Differential Transformer) sensor accounting for the displacement of the top platen inside the triaxial cell, especially during isotropic loading, to estimate the global axial strain $\varepsilon _{a} = \Delta h / H_{0}$ where $H_{0}$ is the initial sample height. The change in sample volume due to a change in the amount of water $\Delta v$ (out of or into the sample) during this process is used to estimate the global volumetric strain $\varepsilon _{v} = \Delta v / V_{0}$ where $V_{0}$ is the initial sample volume and to estimate the solid fraction $\Phi$ (the volume of solids to the total volume). One complementary piezoelectric accelerometer (PCB 607A11) with a resolution of 350 $\mu$g within 10 kHz is used to measure the instantaneous vertical acceleration of the sample top cap.

To follow the fast dynamics of the slip phase in detail, two dynamic data acquisition boards NI 4472B of National Instruments are used concurrently to synchronously acquire various measurements at 10 kHz.

**Figure 3:**
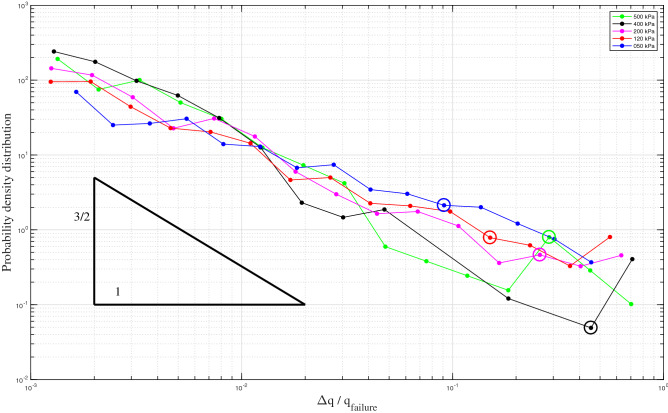
Probability density distributions of deviatoric stress drop $\Delta q$ normalized by $q_{f}$ for various constant confining stresses $p_0$, from 50 up to 500 kPa, following the simple power-law of 3/2 (triangle). The largest saturated laboratory stick-slip motions have $\Delta q/q_{f} \geqslant$ 0.7, approaching the limit value of 1. Large hollow circles signal the particular case of the first stick-slip events with large deformations. The results are binned logarithmically.

**Figure 4:**
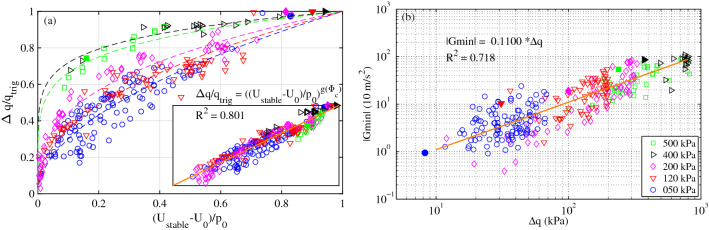
Effects of normalized stabilizing excess pore pressure $(U_{stable}-U_0)/p_0$ and solid fraction $\Phi _{c}$ at constant confining stress from 50 up to 500 kPa on (**a**) normalized deviatoric stress drop $\Delta q/q_{trig}$ with linear scaling relation $g(\Phi _{c}) = a_g*\Phi _{c} + b_g$ ($a_g$ = -15.62, $b_g$ = -9.77, R$^2$ = 0.801). First stick-slip motions (solid symbols) are special cases of strong liquefaction potential. Insets show the unique master curve with the scaling parameter as a function of the initial solid fraction at the beginning of the shearing phase $\Phi _{c}$. (**b**) Linear relations between the maximal vertical acceleration of the sample top cap and deviatoric stress drop.

**Figure 5:**
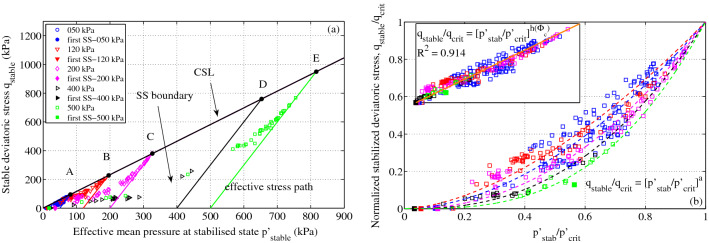
(**a**) Stabilized effective stress state of stick-slip motions in the effective stress ($p'-q$) plane for different constant confining stresses $p'_0$, from 50 (A) up to 500 kPa (E). Inclined solid lines are the usual effective stress paths for drained compression tests from $p'_0$ to the failure point on the critical state line (CSL). Dotted points are the stabilized effective stress state calculated from the stabilized excess pore fluid pressure. (**b**) Normalized stick-slip boundary by failure point on the critical state line. The inset shows the unique stick-slip boundary with the linear scaling relation $h(\Phi _{c}) = a_h*\Phi _{c} + b_h$ ($a_h$ = 53.23, $b_h$ = -29.96, R$^2$ = 0.914).

## Experimental results

In this study, we performed two-step triaxial tests on cylindrical samples made of soda-lime spherical glass beads (mean grain diameter 0.723 mm) (see the SI for details) at five different confining pressures: 50, 100, 200, 300, 400 and 500 kPa. Note the relatively high normal stress, up to 500 kPa, compared to that possible with a slider frictional machine^42,43^ or rotary shear apparatus^9,10^. In the following, we focus our analysis on the representative case of an experiment performed at 400 kPa (Fig. 1). Indeed, all the other experiments we performed exhibit the same quantitative evolution (Fig. S2).

### Isotropic compression collapses

In typical isotropic compression tests performed on real granular materials under fully drained conditions, one expects a continuous increase in the solid fraction $\Phi$ with increasing effective stress $\sigma$^44^. However, with model granular media (such as spherical beads), sawtooth behavior is caused by irregular events (not periodic events) occurring at random triggering effective stresses. In our specific case, we observe 3 successive events occurring at effective stresses ${\sigma }$ of 51, 160 and 293 kPa (Fig. 1b). Each event is characterized by three macroscopic parameters, occurring simultaneously: a sudden rise in the solid fraction $\Delta \Phi$, indicating a jump in compaction volumetric strain $\Delta \varepsilon _{v}$, a jump in axial strain with contraction $\Delta \varepsilon _{a}$ (shown in Fig. 2); and an equally fast reduction in $\sigma$ due to the spontaneous outburst of pore fluid pressure $\Delta U$ occurring at constant solid fraction, followed by a slow recovery of $\sigma$. Since the cylindrical shape of the sample is preserved, these events are referred to as local stick-slip-like isotropic collapses^45^.

This first mandatory step of simple isotropic compression is often overlooked by granular scientists in the study of stick-slip phenomenon in triaxial compression whereas it is of prime interest. Indeed, the existence of random events makes it impossible to fully control the solid fraction $\Phi _{c}$ of model granular materials at the end of this first isotropic loading step and thus at the beginning of the subsequent triaxial compression.

### Stick-slip motions under triaxial compression

Subsequent triaxial drained compression at a constant cell pressure and constant axial strain rate (but at a random $\Phi _{c}$) presents numerous large quasiperiodic patterns in both the stress–strain behavior (Fig. 1c) and change in solid fraction (Fig. 1d). These patterns are often called stick-slip events in friction experiments. The solid lines in Fig. 1 correspond to the high temporal resolution data gathered with our experimental setup. These data accurately reveal both new and complex dynamics during the slip phase (more details are provided in Fig. S4). In contrast, the dashed lines, which represent the quasistatic response of stick-slip motion induced under a compressive loading and drained conditions consistently observed in the granular literature, do not succeed in capturing these complex dynamics. Indeed, at a low acquisition rate, only very few measurement points can be recorded during the slip phase (i.e., between points B and D, in the inset of Fig. 1d).

A stick-slip event is characterized by an abrupt deviatoric stress drop, followed by a more gradual hardening behavior that is interrupted by the next event (Fig. 1c). After the first stick-slip event, these quasiregular stresses drop to a well-defined residual deviatoric stress. Large stress drops can be translated into a large loss of the static frictional coefficient $\Delta \mu$ with $\mu = q/p$ and where *p* is the effective mean stress. The frictional drop $\Delta \mu$ can reach 1.209 for SS$_7$, which is one of the largest reductions observed in laboratory frictional experiments. A stick-slip event is also characterized by a synchronized increase in the solid fraction (volumetric compaction), followed by a more gradual dilating behavior (Fig. 1d). The oscillating behavior in $\Phi$ approaches the maximum packing fraction (0.639) for an assembly of monodisperse spherical particles^46^. However, it never crosses this limit.

The first stick-slip event has a very large deviatoric stress drop $\Delta q$ of 373 kPa, reaching the isotropic state ($q = 0$) and an unusually large incremental axial strain (3.523% in one single step in less than one second). We report, for the first time, the existence of such unconventional stick-slip events with complete loss of friction and $\Delta \mu$ = 0.763, associated with large deformation and potentially leading to a new case of dynamic liquefaction failure due to stick-slip under compression^39^. These extreme cases of vanishing frictional stress were uncommon for stress-hardening materials in granular mechanics. The previously measured simultaneous jumps of $\Delta \varepsilon _{a}$ and $\Delta \Phi$ (or $\Delta \varepsilon _{v}$) between two consecutive stick-slip motions in the granular literature are now interpreted as fast and continuous changes from one steady state to another^6,9^.

Visually, the absence of strain localization was noticed for all the stick-slip events, which can be associated with the diffuse instability phenomenon of loose granular materials^18,22,47^.

These new observational results on fully fluid-filled granular media contrast sharply with the usual results on dry materials with much smaller stress drops and little variation in axial and volumetric strains with numerous quasiperiodic stick-slip events^9,42,48,49^.

### High-temporal-resolution measurements

For a typical stick-slip event occurring at the critical deviatoric stress plateau and large strains, Fig. 2 presents, in blue, the high-temporal-resolution evolution of the top cap acceleration *G*, the deviatoric stress *q*, the excess pore pressure $\Delta U$, the incremental axial strain $\Delta \varepsilon _{a}$ and the incremental volumetric strain $\Delta \varepsilon _{v}$. A semilogarithmic time scale is used to examine the behavior spanning five orders of magnitude while emphasizing the short time scale. The time origin is shifted to the beginning of the changes in acceleration data ± 0.1 ms, which is the current time resolution. Figure S3 shows that all the stick-slip events for the experiment at 400 kPa share the same global behavior as that presented in Fig. 2.

For the seventh stick-slip event presented in Fig. 2, we observe that the first parameters that significantly and quasisimultaneously evolve are the top cap acceleration and the deviatoric stress.

In Fig. 2a, the first event in the acceleration evolution is always negative (up to 100 times higher than the gravity acceleration), and it is therefore representative of a sudden fall of the sample top cap. This first modification of the top cap acceleration always precedes that of the pore pressure by an average of 3.9 ms for the whole set of experiments we performed, with 50–500 kPa of confining stress (see Fig. S7).

In Fig. 2b, the deviatoric stress drops sharply from an uncontrollable triggering value of 823.1 kPa to a well-defined constant residual stress of 74.6 kPa in less than 10 ms. During this 10 ms transient phase, the deviatoric stress oscillates, leading to a brief period with negative deviatoric stress. Nevertheless, the sample does not lose its stability. We suspect that the extremely short duration of this phase is not long enough to fully destroy the granular structure. After this transient phase, the deviatoric stress reaches a steady value, which is maintained for more than 10 seconds before increasing again.

In Fig. 2c, after 5 ms, due to deviatoric stress and vertical acceleration, the pore fluid pressure changes from the imposed constant back pressure $U_{0}$. Two phases characterize this sudden change. First, a transient phase (*I*^39^) in which the pore fluid pressure instantaneously rises and fluctuates like an oscillating underdamped system toward a stabilized value within 300 ms; then, a slow dissipation phase (*III*^39^) in which the pore fluid pressure decays slowly toward its steady-state value of $U_{0}$. The special intermediate phase *II* at constant *U* over a duration larger than one second observed for the specific liquefaction case^39^ not observed here.

In Fig. 2d,e, after a longer time delay of 10 (30) ms, $\Delta \varepsilon _{a}$ ($\Delta \varepsilon _{v}$) begins to continuously change from one steady-state to another. This particular stick-slip event has a very fast axial strain rate of approximately 0.461%/s (0.212%/s), contrasting with the constant prescribed rate of 0.0048%/s for drained compression loading used to represent complete drainage. The instantaneous axial strain rate is even larger, at approximately 4.0%/s. The first stick-slip event has the largest instantaneous axial strain rate, up to one decade larger at 36.5%/s. This results in a dynamic regime for the slip phase, with a high dimensionless inertial number^44^ ($I \approx$ 10$^{-3}$), compared to that of the quasistatic regime ($I \approx$ 10$^{-6}$) of the stick phase. Transient phase *I* essentially occurs under undrained conditions (no volumetric variation) before 40 ms, with very small volume changes below 0.01%. The observed time delay of $\Delta \varepsilon _{a}$ and $\Delta \varepsilon _{v}$ probably results in the inability of the displacement sensors (due to insufficient sensitivity) to record the earlier changes in very small amplitude.

On Fig. 2, we also represent, in red, the evolution of the excess pore pressure $\Delta U$, the incremental axial strain $\Delta \varepsilon _{a}$ and of the incremental volumetric strain $\Delta \varepsilon _{v}$ for a typical instability event under isotropic compression. The very similar qualitative evolutions of these parameters with those previously described may suggest that the two corresponding event types share the same physical origin despite a different external loading (isotropic stress-driven versus triaxial strain-driven compression).

The uncommon use of a semilogarithmic time scale is justified in our experiment by the complex dynamics of the stick-slip events that not only have a relatively long duration (a few seconds) but also exhibit very high-frequency phenomena at the beginning. Indeed, on a linear time scale (Fig. S4) the global dynamical behavior (i.e., a rapid decrease followed by a slow recovery of the deviatoric stress) is confirmed but reveals much less detail of the beginning of the slip event. Moreover, Fig. S4 again emphasizes the importance of the frequency of data acquisition (solid lines versus dotted lines) to capture the complexity in the slip phase.

With the same high-resolution data from qualified sensors, Fig. S3 shows that the whole set of stick-slip events under 400 kPa presents the same qualitative evolution as those described for the one specific stick-slip event in Fig. 2. Moreover, we obtained identical results^50^ (not presented here) for all the tested confining pressures ranging from 50 to 500 kPa. From all the collected high-resolution measurements (cf. Fig. 2), we propose a scenario to describe the sequence of events that occur during stick-slip motion.

- First, the systematic and simultaneous changes in deviatoric stress and top cap acceleration suggest that a fast underlying modification of the granular microstructure occurs. This modification of the granular sample allows the top cap to settle (this top cap is not physically attached to the vertical loading device). This modification might be due to local changes in the force chain network^51–53^, followed by either ejection of some grains or local rearrangement, such as the one described in the shear transformation zone (STZ) framework^54^ facilitated by the anisotropic granular structure, the spherical grain shape and the incompressibility of the pore water in the fully saturated assembly. Recent X-ray imaging of dry granular assemblies of various particle shapes also strongly suggests local particle rearrangements as a plausible physical cause for stick-slip phenomena under low confining stress^49^.
- Second, the modification of the granular structure briefly and sharply increases the pore pressure. As a consequence, the Terzaghi effective stress^55^ is significantly reduced, and the grains are locally separated, allowing slip phases with larger axial strains than that observed in the dry case^17,35,36,38^. Indeed, the small and positive delay with respect to $\Delta q$ rules out the possibility of pore pressure as the main cause for the stick-slip phenomenon. The consistent positive delay of pore pressure outburst also indicates a counterintuitive uncoupled response between the solid and liquid phases for a fully saturated two-phase mixture (Fig. S7). Since pore pressure generation and dissipation occur under a constant deviatoric stress $q_{stable}$, the new dynamic slip phase can be interpreted as dynamic consolidation^56^ at a constant $q_{stable}$ (Fig. 1c; Fig. S1) with a constant dynamic consolidation coefficient $C_s^{Dyn}$, regardless of confining stress (Fig. S6). The total duration of this dynamic consolidation is less than a few seconds. This dynamic consolidation is an essential feature of stick-slip motion under compression in saturated granular materials. The presence of the fluid phase and its pressure outburst seem to be responsible for the large incremental axial and volumetric strains that are observed in this study but not in studies of dry cases.
- Last, following pore pressure dissipation, the grains reconnect in the stick phase to form a new and more compact structural rearrangement^57^. Thus, the loading device contacts the top cap and allows the rearranged granular sample to be loaded again. This increase in the deviatoric stress (and the solid fraction) is the beginning (stick phase) of the following event. One can note in Fig. 1c, that the solid fraction oscillates up to a stable value close to the packing limits of 0.63.

## Analysis and discussion

### Stress drop magnitude distributions

In Fig. 3, the probability density distributions of the magnitude of the normalized deviatoric stress drop $\Delta q/q_{f}$ in logarithmic space follow a simple power-law distribution $P(s) \sim s^{-\tau }$. The stress drop is normalized by the deviatoric stress at the critical stage $q_{f}$ determined by the Mohr-Coulomb failure criterion for cohesionless materials in soil mechanics^58^ while ignoring the stick-slip events. Irrespective of the confining pressure up to 500 kPa, the distributions for laboratory stick slips for $\Delta q/q_{f} \geqslant$ 0.001, approach the traditional Gutenberg–Richter law for earthquakes. In our specific experimental conditions, i.e., saturated granular packing with a high confining pressure, the presence of very large stick-slip motions ($\Delta q/q_{f} \geqslant$ 0.7) to approximately the isotropic stress level ($\Delta q/q_{f}$ = 1.0) allows the power law to span over more than 3 decades with no upper cutoff. The power law exponent $\tau \approx$ 1.33 was found to be independent of $p_0$, approximating the usual value from mean field theory of 3/2 (a triangle serves as a visual guide in Fig. 3)^8^. As a comparison, in Murphy^49^, with spherical beads under dry conditions and only 20 kPa of confining pressure, found a truncated power law over less than two decades with a similar exponent ($\tau \approx$ 1.4) but with a very small cutoff ($\approx$ 0.004).

The very special case of the first stick-slip with vanishing residual frictional stress and the strongest liquefaction potential ($q = 0$, large hollow circles) has $\Delta q/q_{f}$ ranging from 0.08 to 0.42. Counterintuitively, the most destructive stick-slip, in terms of irreversible damage of the granular structure, is not caused by the most powerful event in terms of mechanical energy. After the first stick-slip, less than one-tenth of the failure stress $q_f$ is enough to completely destroy the granular structure in a rare diffuse liquefaction failure^59^.

### Quasideterministic nature

In the evolution of the pore pressure (see Fig. 2c), if the duration of the quasistatic stabilized excess pore pressure $U_{stable}$ exceeds more than one second, catastrophic failure arises, even sometimes for the surprising case of isotropic compression^39^. According to classical granular physics, this isotropic liquefaction should be conceptually impossible. This is the main reason to explore the effects of $U_{stable}$ on stick-slip events.

Figure 4a shows the evolution of the normalized deviatoric stress drop $\Delta q/q_{trig}$ as a function of the normalized stabilized excess pore pressure $(U_{stable}-U_0)/p_0$ from a series of compression tests under constant cell pressures spanning from 50 to 500 kPa. $\Delta q$ (respectively $\Delta U$) is normalized by $q_{trig}$ (respectively $p_0$) to remove the effects of triggering stress (respectively cell pressure) on the stick-slip behavior. On a log-log scale, all the curves follow a single linear master curve which proves the existence of an empirical power law between these two parameters. The single scaling parameter of these relations depends solely and linearly, $a*\Phi _{c} + b$, on the initial solid fraction at the beginning of the shearing phase $\Phi _{c}$, hence permitting the uncoupling of the role of $p_0$ and $\Phi _{c}$, as usual in soil mechanics^60–62^.

The first stick-slip motions present the strongest liquefaction potential and the largest friction drop because the normalized excess pore pressure is larger than 0.8 (solid symbols in Fig. 4a and Fig. S5), and the deviatoric stress vanishes. $(U_{stable}-U_0)/p_0$ = 1 and $\Delta q/q_{trig}$ = 1 (isotropic stress state) are among the necessary conditions for liquefaction, as the gaps that would result in an extreme case of failure^39^ are filled due to very large axial and volumetric deformations.

The deviatoric stress drop $\Delta q$ scaled linearly with the amplitude of the first peak of the vertical acceleration $G_{min}$ over nearly two decades (Fig. 4b). Assuming that $G_{min}$ is mainly induced and thus representative of granular rearrangement events, this linear relationship, with a correlation coefficient of 0.7, suggests the quasideterministic nature of granular stick-slip motion. Indeed, the macroscopic stick-slip behavior ($\Delta \varepsilon _{a}$, $\Delta \varepsilon _{v}$ and $\Delta q$) is mostly determined by short-lived granular rearrangement events with durations of less than one millisecond. The greater the magnitude of an event is, the greater the probability of instability and the greater the magnitude of the deviatoric stress drop.

A practical consequence of these experimental findings is that stick-slip behavior cannot be controlled in a laboratory setting for two main reasons. First, the stick-slip behavior is mainly driven by the initial solid fraction $\Phi _{c}$ which is impossible to control due to the presence of random events during the isotropic compression (see Fig. S2). Second, once initiated, the duration of the initial instabilities is less than a few seconds in saturated materials. Unfortunately, the local triggering mechanisms of granular rearrangement remain unknown.

### Unique stick-slip boundary

Figure 5a shows the temporarily stabilized effective stress state at the end of the deviatoric stress drop (hollow symbols) in the effective stress plane ($p-q$). Due to the fast pore fluid outburst, these short-lived stress states temporarily leave the effective stress path of drained compression (inclined solid lines) and return to the same effective stress path following pore pressure dissipation. The above double normalization by $p_0$ and $\Phi _{c}$ clearly reveals a unique stick-slip boundary in Fig. 5b with appropriate scaling parameters from the failure point ($q_{f}$) on the critical state near the liquefaction point t (*q* = 0) in the normalized effective stress plane. All the first slick-slip events (solid symbols) under the different confining stresses are located near the liquefaction point at the origin of the effective stress plane. This observation can explain the inability of slick-slip events induced under compression to liquefy, except for the first slick-slip events, and the frequent occurrence of isotropic liquefaction as special cases of slick-slip with no deviatoric stress.

This unique stress boundary strengthens the quasideterministic nature of the stick-slip phenomenon.

### Appearance conditions

To the best of our knowledge, in granular mechanics, natural granular materials (i.e., sands) never experience such dynamic instabilities. However, these laboratory stick-slip events did occur for model granular materials under triaxial compression below 1 MPa regardless of drainage (undrained or drained), loading (isotropic stress-driven or triaxial strain-driven), saturation (dry, wet or saturated), particle size distribution (mono- or polydisperse), particle shape, mixture conditions (sand and glass beads) and pore fluid viscosity (air, water or oil)^17,35–38,40,49,63–66^. These events can also be observed during 2D shear^66–68^ and in double direct shear experiments^69–71^ under large confining stresses exceeding 1 MPa to simulate tectonic fault gouges.

Experimentally, these large dynamic instabilities may appear under some specific conditions: (1) an initially structural anisotropic state created by the moist tamping fabrication technique and revealed by a high anisotropic coefficient during isotropic drained compression^59^, (2) an initially loose threshold solid fraction at 20 kPa of confining stress with random loose packing, and (3) an axial strain rate below the critical axial strain rate (critical velocity in^6,72^) during triaxial drained compression^17^.

Furthermore, these laboratory experiments overlook the mandatory step of isotropic compression preceding triaxial compression and the paramount role of pore fluid pressure in fluid-saturated instability events. Excess pore fluid pressure forms very quickly, probably due to the many valuable features of stick-slip dynamics overlooked during such experiments. As in situ instrumentation has measured a similar surge in excess pore pressure in liquefied soils during recent earthquakes^73–75^, it deserves further attention as a possible indicator of saturated stick-slip events. Many previously unknown details of the missing features are revealed in this high-temporal-resolution study.

## Conclusions and perspectives

In a recent series of careful laboratory experiments using triaxial equipment, we detected, with a high level of confidence, a new spectrum of dynamic instabilities in saturated granular materials. These instabilities include unconventional observed behaviors such as irreversible damage in isotropic compression, termed isotropic collapse, up to the destruction of the granular skeleton, termed isotropic liquefaction^39,45^. In this paper, we extend the range of the observed spontaneous behaviors in triaxial compression and discover some new facets of stick-slip phenomena, including some of the largest laboratory earthquakes with the highest observed frictional stress drop to date and even, surprising, rare liquefaction during first stick-slip events under moderate stress loadings.

Our results indicate the quasideterministic nature of granular instabilities (isotropic collapse and stick-slip motion under compression) through simple scaling laws with uncoupling effects of the initial cell stress and solid fraction. We show that the magnitude of the laboratory earthquake-like event is conditioned by the magnitude of the short-lived vertical top cap acceleration in the first few milliseconds. These phenomenological scaling relations demonstrate the ability to predict the macroscopic mechanical parameters of laboratory earthquakes from the characteristics of the beginning of an instability event. Additionally, these mechanical parameters are essentially controlled by the stabilized excess pore fluid pressure during a dynamic consolidation process at a constant deviatoric stress. However, this pore fluid outburst is definitively not the causative mechanism of the observed instability with repeated rebuilding of the granular structure but is more likely an important consequence thereof.

Two basic ingredients are required for the appearance of these dynamic instabilities on a fully saturated granular assembly: an initially loose density structure associated with an initially anisotropic strain state. Although further experiments are necessary to fully assess the deterministic nature of these events, the hypothesis of microstructural changes is one of the leading hypotheses presented to describe the physical triggering mechanisms.

Even though these new stick-slip dynamics do not solve the mysteries surrounding the unpredictable triggering stress or the unexpected liquefaction under isotropic compression, they may improve the understanding of the causes of granular instabilities during earthquakes in fluid-saturated conditions both in a laboratory and in nature. Furthermore, they present a new set of challenges for scientists studying geomaterials with theoretical and numerical approaches.

The results of this experimental study on spherical glass beads, often used as analogue materials for fault gouge, can be extended qualitatively to gain a better understanding of earthquake mechanics. They confirm the paramount role of pore fluid pressure outbursts as one of the key mechanisms for tectonic fault slip via reducing the normal effective stress^71,76^, with some new perspectives. This pore fluid overpressure can be created artificially with deep wastewater injection^77,78^, or controlled with a laboratory apparatus^71,79^. The uncontrolled triggering stress observed in this work can create spontaneous pore fluid pressure outbursts approaching lithostatic stress conditions and the largest laboratory earthquakes to date, with devastating liquefaction failure in less than one second. Our data can progress the exploration of the quasideterministic nature of natural earthquakes.

## Supplementary Information

Supplementary Information.
