# Publisher Correction: High-temporal-resolution quasideterministic dynamics of granular stick-slip

**DOI:** 10.1038/s41598-021-95571-w

**Published:** 2021-08-04

**Authors:** T. T. T. Nguyen, T. Doanh, A. Le Bot, D. Dalmas

**Affiliations:** 1 Ecole Nationale des Travaux Publics de L’Etat, LGCB, LTDS (UMR 5513), Vaulx en Velin, France; 2 Ecole Centrale de Lyon, LTDS (UMR 5513), Ecully, France

Correction to: *Scientific Reports* 10.1038/s41598-021-82581-x, published online 03 February 2021

The original version of this Article contained errors.

In the legend of Figure 1,

“Fig. (S4 for additional details).”

now reads:

“(Fig. S4 for additional details).”

Additionally, in the Experimental results section, under the subheading ‘Isotropic compression collapses’,

“In typical isotropic compression tests performed on real granular materials under fully drained conditions, one expects a continuous increase in the solid fractio Φ with increasing effective stress σ^44^.”

now reads:

“In typical isotropic compression tests performed on real granular materials under fully drained conditions, one expects a continuous increase in the solid fraction Φ with increasing effective stress σ^44^.”

Finally, the Author Contributions section,

“H.J.O and B.J.Y constructed the original idea according to concept of analysis. D.K.K and H.J.O conducted experiments. H.J.O, Y.C.C and W.G.H carried out the characterizations. H.J.O wrote the manuscript. S.J.L, J.B.J were involved in the piezoelectric study of using the PLLA/BaTiO_3_ fibres and textile sensors. B.J.Y and H.K, J.H.K, W.G.H and S.W.K confirmed experimental result and discussed with H.J.O. All authors contributed and commented on this manuscript.”

now reads:

“All authors contributed equally to this work.”

The original Article has been corrected.

