# Supplementary material for: High-temporal-resolution quasideterministic dynamics of granular stick-slip
Source: Sci Rep. 2021 Feb 3;11:2902. doi: 10.1038/s41598-021-82581-x (PMC7858602; doi:10.1038/s41598-021-82581-x)
Supplement: Supplementary file 1 — Supplementary Information. [file 41598_2021_82581_MOESM1_ESM.pdf]

High-temporal-resolution quasideterministic dynamics of granular stick-slip

T.T.T. Nguyen[1], **T. Doanh**[1,*], **A. Le Bot**[2], **D. Dalmas**[2]

[1] Ecole Nationale des Travaux Publics de l'Etat. LGCB, LTDS (UMR 5513). Vaulx en Velin. France.
[2] Ecole Centrale de Lyon. LTDS (UMR 5513). Ecully. France.
[*] thiep.doanh@entpe.fr

Supplementary Information

Experimental details

For this study, we chose industrial soda-lime glass beads "Sil-glass" commercialized by CVP, Linselles, France (www.Cvp-abrasif-broyage.com). These perfectly spherical glass beads SLG 6-8 are often used in laboratory testing as a proxy for Hostun and Toyoura sands[1,2]. These beads are characterized as a clean and poorly graded fine-grained material (mean grain diameter, D_{50} = 0.723 mm).

For this triaxial setup (Fig. 1a), all the samples with a short cylindrical shape, H_0 = 70 mm in height and D_0 = 70 mm in diameter, are fabricated using a modified moist tamping and under compaction method[3,4]. This fabrication process is usually employed in geotechnical laboratories to create loose and contractive granular samples in three steps. First, predetermined quantities of moist glass beads are mixed with 2% distilled water by weight, and then the mixture is placed in the triaxial mold in five layers inside an open-ended cylindrical latex membrane with a thickness of 0.3 mm. Then, each layer is gently compacted up to the prescribed thickness using a flat-bottom circular tamper made of stainless steel 20 mm in diameter. An enlarged top cap finally seals the sample, and a small vacuum of only 10 kPa is applied to stabilize the granular sample during the removal of the cylindrical mold and the installation of internal sensors inside the triaxial cell. The initial solid fractionn Φ_0 at the end of the fabrication state is estimated from the sample dimensions. Last, the triaxial cell is filled with water, and the previous vacuum is replaced by a cell pressure of 20 kPa.

A saturation process is applied next in three steps to obtain a high Skempton's coefficient B = $\Delta U/\Delta\sigma \geqslant 0.95$ and a fully saturated sample[5]. The CO_2 method[6] under a very low gradient of only 0.2 kPa is used for at least 1 hour before flushing deaired and distilled water, followed by the application of a constant back pressure U_0 of 200 kPa.

Additionally, the solid fraction at the end of the triaxial test was also carefully measured from the final water content to back-calculate and verify the solid fraction Φ_c at the beginning of the triaxial test[7].

Technically, in the first step of isotropic compression under the stress-controlled mode with a constant stress rate, the cell pressure is slowly applied using compressed air ($\sigma_r = \sigma_3 = \sigma_2 = \sigma_1$, where σ indicates a principal stress and σ_r the radial stress), to a desired final value $\sigma_r = p_0$, which is the prescribed final cell pressure. Once reaching p_0, if the sample is still geometrically stable, the second step of triaxial compression under the strain-controlled mode with a constant axial strain rate and constant cell pressure begins by increasing only the axial (major principal) stress $\sigma_a = \sigma_1$ until until failure while maintaining a constant radial stress $\sigma_r = \sigma_3 = \sigma_2 = p_0$. The deviatoric stress q is defined as $q = \sigma_1 - \sigma_3 = \sigma_a - \sigma_r$, and the mean pressure $p = (\sigma_1 + 2 * \sigma_3)/3$. Note that the isotropic compression step corresponds to a deviatoric stress of zero..

For a fully saturated granular mixture, we use the intergranular stress σ' following the Terzaghi effective stress principle for a two-phase solid-fluid mixture $\sigma' = \sigma - U$, where σ is the total stress, and U is the pore fluid pressure[8]. Since the pore fluid pressure is constant in drained conditions, we can omit the prime superscript for the effective stress in this paper without compromising the clarity.

All the measurements in the event signal were smoothed using a moving average filter and Savitzky-Golay filter of 7th order to remove electrical noise and powerline interference at 50 Hz.

Fig. S1 presents the schematic temporal evolution of deviatoric stress q and pore pressure U during the laboratory stick-slip experiments to clarify the essential notations used in the main text. The logarithmic scale of time is proposed to show the sudden drop in q and the spontaneous rise in U in detail. The origin of time begins at the abrupt drop in q, which was manually detected by eye since the automatic detection procedure was far more difficult than expected. The deviatoric stress drop Δq is estimated from q_{trig} to q_{stable}, while ignoring the first minimum q_{vib} of the transient phase. The stabilized excess pore pressure ΔU_{stable} calculated from constant back-pressure U_0 to the stabilized value U_{stable}, also ignores the short-lived peak U^{peak}. The excess pore pressure is further normalized by ΔU_{stable} to eliminate the eventual dependence on ΔU_{stable}. Isotropic experiments have no deviatoric stress.

Stick-slip dynamics

Fig. S2 shows the stick-slip behavior when varying the confining stress from 50 to 500 kPa, including the first step of isotropic compression. Pore fluid outbursts systematically occur for each stick-slip event and then return to a normal level of constant

back pressure. Isotropic compression up to 500 kPa during the triaxial compression test at 500 kPa results in only one single collapse at 96 kPa, and the shear behavior reflects three exceptional stick-slip motion SS_1, SS_4 and SS_{25} with large incremental axial and volumetric strains. This test paves the way for the strong possibility of stick-slip liquefaction beyond the first stick-slip event, which has never been observed before in triaxial compression drained experiments. The first stick-slip event always causes the stress to drop to the isotropic stress level, confirming its large liquefaction potential and the complete dissipation of the accumulated energy.

The lack of instability events in the isotropic compression phase of tests below 120 kPa signals the unpredictability of these events, since isotropic collapses and even liquefaction can occur well below 100 kPa in repeated experiments[2,9].

All large stick-slip motions under 400 kPa of confining stress exhibit the same type of temporal evolution (Fig. S3). In this figure, the stick-slip motions are identified by their number in the right-hand color bar with increasing axial strain at the beginning of the stick-slip event. Note the fast drop time of q below 2 ms, the same dominant frequency of U, the rapid and continuous development of $\Delta\varepsilon_a$ and $\Delta\varepsilon_v$, with the exception of the largest final values for the first stick-slip event, and the occurrence of a small, although distinctive and unmistakable, extension (negative) axial strain in numerous stick-slip events. The tilt of the sample top cap due to local instability can offer an acceptable explanation for these abnormal measurements.

The deviatoric stress drop always preceded the quick generation of global pore fluid pressure by approximately 5.0 ms and the macroscopic axial and volumetric strains by more than 10 ms in this series of tests.

These observations rule out the possibility of pore fluid pressure as the main physical triggering mechanism of stick-slip motion and strengthen the above hypothesis of microstructural changes.

The separation of the excess pore pressure evolution into two phases is still valid, with a clear division at 300 ms. Furthermore, the similarity of the pore pressure records, superimposed on the results of isotropic collapse I_C (thick magenta line), as well as the evolution of the macroscopic strains (axial and volumetric), for the two kinds of loading considered (isotropic and triaxial compression) strongly suggest the same causative mechanisms.

Considered together, the global stress-strain and volumetric behaviors are significantly different in Fig. 1, especially the dynamic consolidation at a constant q and the gradual development of axial and volumetric strains in the time between two consecutive stick-slip events. The dashed lines represent the usual behaviors of stick-slip motion under drained conditions and compressive loading in the granular literature, as in Fig.1, ignoring the dynamics of the slip phase. From the data, including reliable pore pressure measurements, these new global behaviors simply include the dynamic nature of the slip phase and the well-known quasistatic response of the stick phase.

Fig. S4 reveals the temporal evolution of a typical large stick-slip event induced by compression in terms of the stress-strain behavior, SS_7 at 10% axial strain in Fig. 1, with a positive q_{stable} using the usual linear time scale. The new dynamic behavior (shown in red) of slip instabilities is quite different from that presented in the granular literature (blue). The inset figures emphasize the short duration of the slip dynamics, less than two seconds. The dynamic slip phase or the deviatoric stress drop BC in Fig. S4a is followed by a much shorter development of axial strain CD than that commonly observed for the first stick-slip event under dynamic consolidation at a constant q_{stable} (approximately 0.5%). The loss of controllability in imposing a constant axial strain rate (inclined dashed upward arrow) is shown in Fig. S4b. The following stick phase DD_1E shows a gradual reconnection with the loading ram, when the granular sample resumes a full quasistatic response at point E. After a very large axial strain rate arises in slip phase BC, the short section DD_1 shows an unexpected decrease in the axial strain rate to zero. The volumetric strain in Fig. S4c largely contracts during slip phase BC and dilates after phase transformation point PT (i.e., the transition from contraction to dilation[10,11]). During this stick-slip event, the granular sample is locally denser than the critical state while paradoxically preserving the global loose density, as shown in Fig. 1.

Overall, these experiments show the loss of controllability in stress in isotropic compression and in strain in triaxial compression at the onset of the instability and the combined effects of void ratio and cell pressure: A loose granular assembly undergoes more stick-slip events, and a large confining stress increases the magnitude of the stress drop significantly, resulting in the largest laboratory stick-slip events observed to date. Paradoxically, the mechanical behavior globally behaves like a loose granular material with a deviatoric stress plateau and volumetric contraction at large strains and locally in a dense state with stress softening and volumetric dilatancy beyond the local phase transformation point PT, where the volumetric behavior changes from contraction to dilation (the dashed line in the 500 kPa experiment)[1].

The similar results for all tests performed in the confining stress range of 50 to 500 kPa strengthen the understanding of the dynamic characteristics of the slip phase.

Dynamic stress path

For each stick-slip event, the effective stress path (ESP) is schematized in Fig. S1. Line GA of slope 3 represents the usual effective and total stress paths for drained triaxial compression, starting from point G at constant cell pressure $\sigma_3' = p_0'$ to the point of failure F at large strains (not shown). From point B of q_{trig}, the deviatoric stress drops sharply to point C at q_{stable}. The instantaneous surge of pore pressure shifts the ESP horizontally to the left to temporary point R at the stabilized excess pore

pressure ΔU_{stable} and constant q_{stable}. Then, the pore pressure dissipation moves the ESP back to point C, signaling the end of the dynamic slip phase and the beginning of the quasistatic stick phase of line CB. The dynamic consolidation RD_1E at constant deviatoric stress q_{stable} has two components: first the dynamic generation of pore pressure of line MR of the transient phase I and then the dynamic pore pressure dissipation of line RD_1 of the dissipation phase III.

This dynamic consolidation is represented by only one parameter in the usual compressibility diagram, the dynamic overconsolidation coefficient C_s^{Dyn}, since the granular sample has a past maximum q_{trig}. A constant C_s^{Dyn} is obtained, except for some small stick-slip motion (Fig. S6). Since $C_s^{Dyn} \approx C_c$ in isotropic compression on virgin material (Fig. S2 top), the model granular assembly has an elastic granular structure, contrasting strongly with the well-known inelastic nature of natural sands.

Uncoupling solid-fluid response

The positive time delay between the normalized deviatoric stress drop and pore pressure generation is fairly consistent for all the stick-slip motions observed, between 2 and 6 ms, regardless of the confining stress in the studied range, except that the test at 50 kPa generated a low excess pore fluid pressure (Fig. S7). The deviatoric stress drop is normalized by deviatoric stress triggering. Owing to the synchronous deviatoric stress and top cap acceleration signals, as noted earlier in Fig. 2, some microstructural changes might occur shortly before any significant modifications of pore fluid pressure or axial and volumetric displacements. This consistently positive time delay indicates a surprising uncoupling response between the solid (granular skeleton) and the pore fluid (deaired water) for all stick-slip motions occurring in the simple two-phase mixture.

The right-skewed histogram (n = 264) suggests a log-normal distribution with a mean time delay of 3.9 ± 1.7 ms, regardless of the cell pressure up to 500 kPa. Some outliers beyond 8 ms belong to small stick-slip motions under a low confining stress of 50 kPa with a small excess pore fluid pressure. The presence of double peaks near 2 and 4 ms suggests that there are other parameters that control this time delay, possibly related to the quasisaturated state despite a high Skempton's coefficient B[12].

References

1. Doanh, T., Hoang, M.T., Roux, J.-N., Dequeker, C. Stick-slip behaviour of model granular materials in drained triaxial compression. *Granul. Matter* **15**, 1–23 (2013).
2. Doanh, T., Abdelmoula, N., Nguyên, T.T.T., Hans, S., Boutin, C., Le Bot, A. Unexpected liquefaction under isotropic consolidation of idealized granular materials. *Granul. Matter* **18**, 67 (2016).
3. Bjerrum, L., Krimgstad, S., Kummeneje, O. The shear strength of a fine sand. In *Proc. 5th Int. Conf. Soil. Mech. Found. Engrg.*, vol. 1, 29–37 (1961).
4. Ladd, R.S. Preparing test specimens using undercompaction. *Geotech. Test. J.* **1**, 16–23 (1978).
5. Skempton, A. W., Taylor, R.N. The pore pressure coefficients A and B. *Geotechnique* **4**, 143–147 (1954).
6. Lade, P.V., Duncan, J.M. Cubical triaxial tests on cohesionless soil. *J. Soil Mech. Found, ASCE* **99**, 793–812 (1973).
7. Verdugo, R., Ishihara, K. The steady state of sandy soils. *Soils Found.* **36**, 81–91 (1996).
8. Terzaghi, K., Peck, R.P., Mesri, G. *Soil Mechanics in Engineering Practice, 3rd Edition* (John Wiley, 1996).
9. Doanh, T., Le Bot, A., Abdelmoula, N., Hans, S., Boutin, C. Liquefaction of immersed granular media under isotropic compression. *Eur. Lett.* **108**, 24004 (2014).
10. Luong, M.P. Etat caractéristique du sol. *Comptes Rendus de l'Académie des Sci. series B* 305–307 (1978).
11. Tatsuoka, F., Ishihara, K. Yielding of sand in triaxial compression. *Soils Found.* **14**, 63–76 (1974).
12. Nguyen, T.T.T. *Dynamic instabilities of model granular materials*. PhD thesis, Ecole Nationale Des Travaux Publics de l'Etat (2019).
13. Adjemian, F., Evesque, P. Experimental study of stick-slip behaviour. *Int. J. Num. Ana. Meth. Geom.* **28**, 501–530 (2004).
14. Çabalar, A.F., Clayton, C. R. I. Some observations of the effects of pore fluids on the triaxial behaviour of a sand. *Granul. Matter* **12**, 87–95 (2010).
15. Wu, K., Abriak, N., Becquart, F., Pizette, P., Rémond, S., Liu, S. Shear mechanical behavior of model materials samples by experimental triaxial tests: case study of 4 mm diameter glass beads. *Granul. Matter* **19**, 65 (2017).

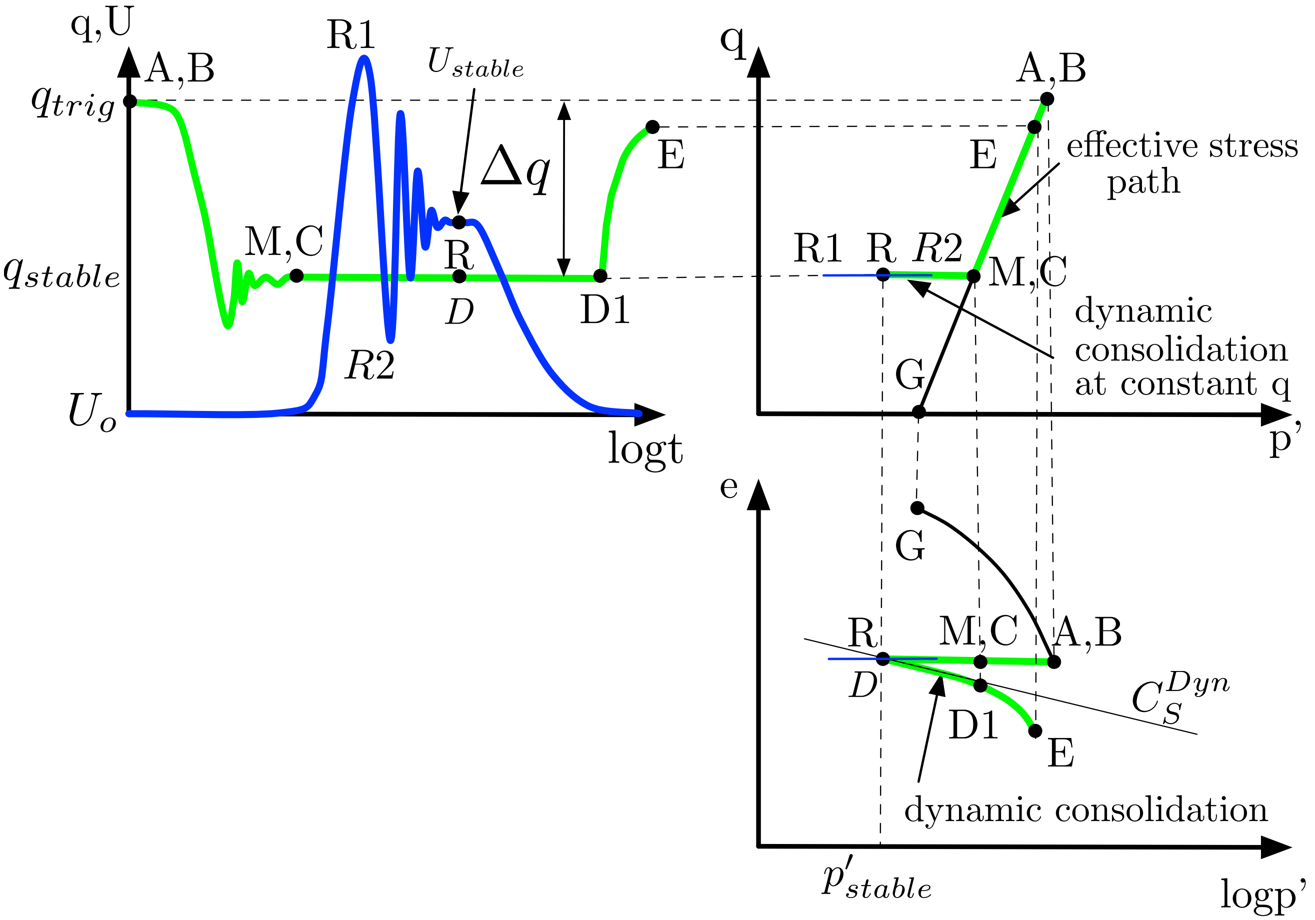

Supplementary Fig. S1. Schematic view of the temporal evolution of deviatoric stress q (green) and pore pressure U (blue), the effective stress path in the effective stress $(p' - q)$ plane starting from an isotropic stress state G of constant effective mean pressure p'_0 and the compressibility plane (e−logp') during stick-slip motions induced by triaxial compression laboratory experiments under drained conditions. Point R_1 gives the excess pore pressure peak, R_2 the minimum of excess pore pressure, and R the position of stabilized excess pore pressure U_{stable} in the temporal evolution of deviatoric stress at well-defined residual frictional stress q_{stable} from A to E: A is at the loading stick phase at a constant q of the critical stage, B is at the beginning of the slip phase at an uncontrolled and unpredictable q_{trig}, C is at the end of stress drop at q_{stable}, D is at the deviatoric stress at U_{stable}, D_1 is at the beginning of the stick phase, and E is on the stick phase, beyond the phase transformation point PT. C_s^{Dyn} is the dynamic consolidation coefficient of the effective stress path DD_1E.

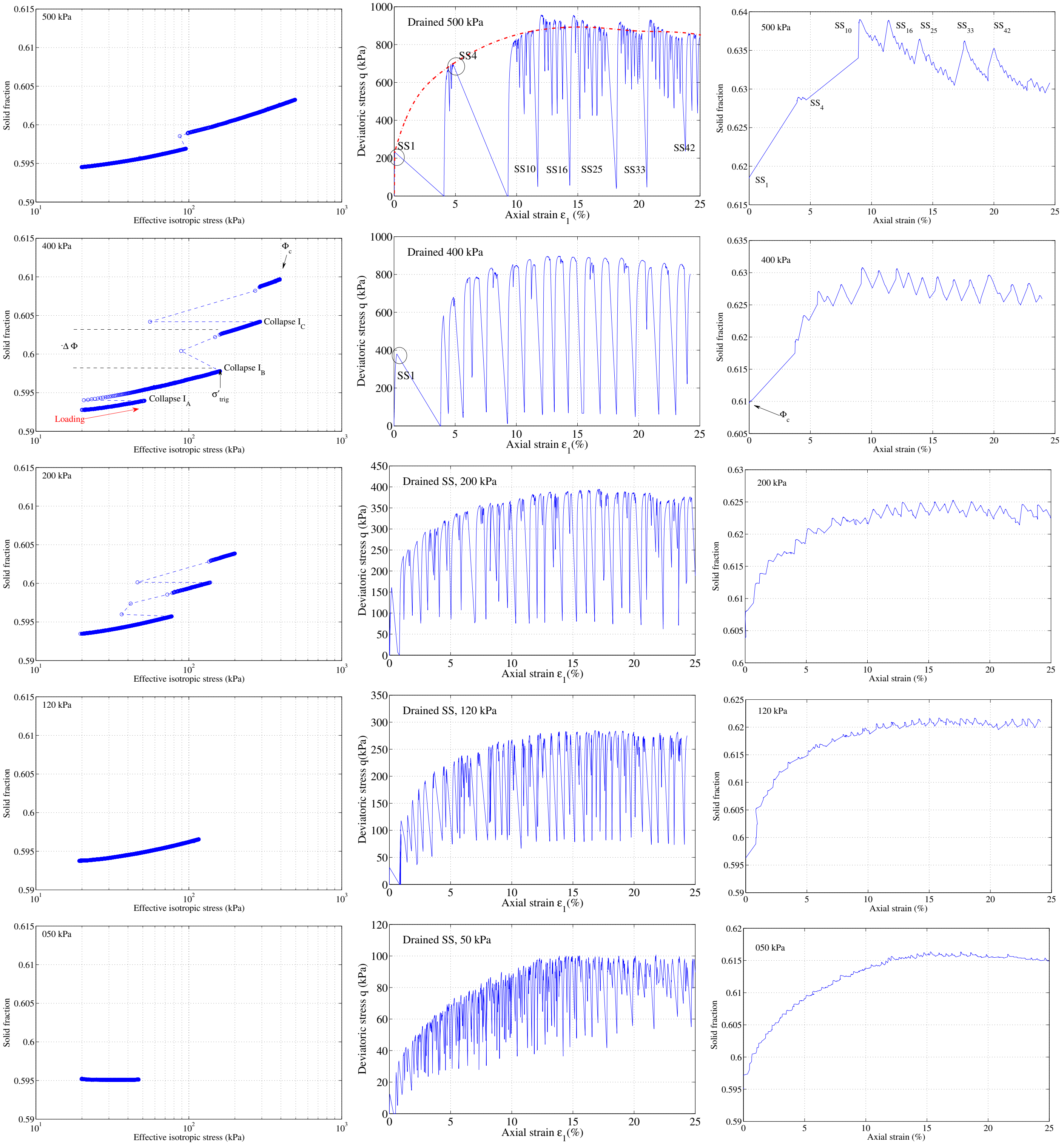

Supplementary Fig. S2. The stick-slip behavior of saturated and loose model granular materials under compression from 500 kPa (top) down to 50 kPa (bottom) of confining pressure using a typical low sampling rate: (left) evolution of solid fraction in the first step of isotropic drained compression to the desired confining stress in the stress-controlled mode, (middle) stress-strain and (right) solid fraction evolutions in the subsequent step of triaxial drained compression in the axial strain-controlled mode. The red dashed lines in the 500 kPa experiment show the hypothetical results without instabilities.

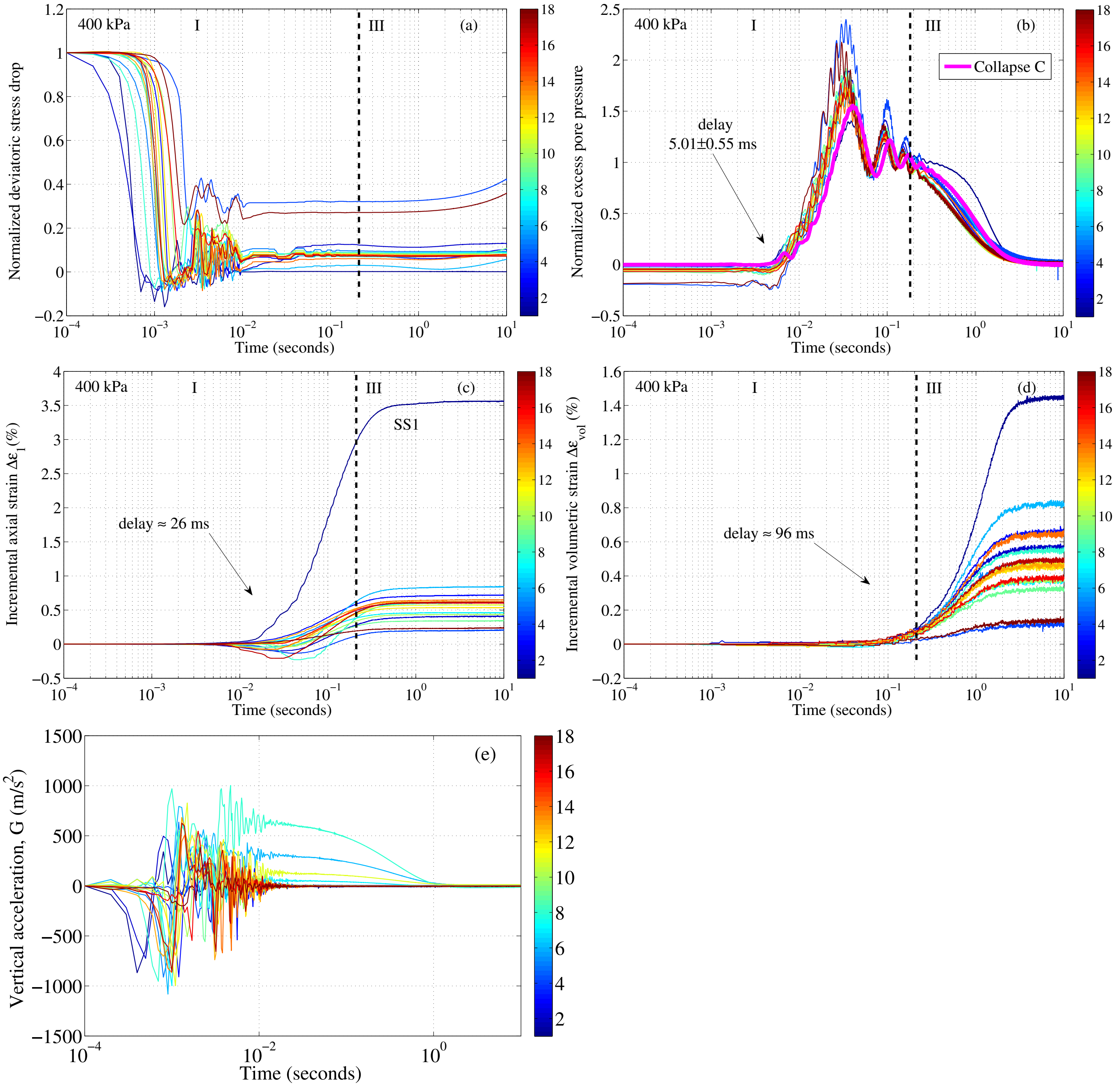

Supplementary Fig. S3. Time evolution of the (a) normalized deviatoric stress drop $\Delta q/q_{trig}$, (b) normalized excess pore pressure $(U-U_0)/U_{stable}$, (c) incremental axial strain, (d) incremental volumetric strain and (e) vertical top cap acceleration during stick-slip events in drained compression tests at 400 kPa of confining stress, up to 20% of the axial strain. The deviatoric stress is normalized by the triggering value at the beginning of the slip event and the excess pore pressure is normalized by its stabilized value after transient phase I. The right color bar gives the index of successive stick-slip events, in terms of the corresponding proportion of the increasing axial strain at the beginning of the stick-slip event.

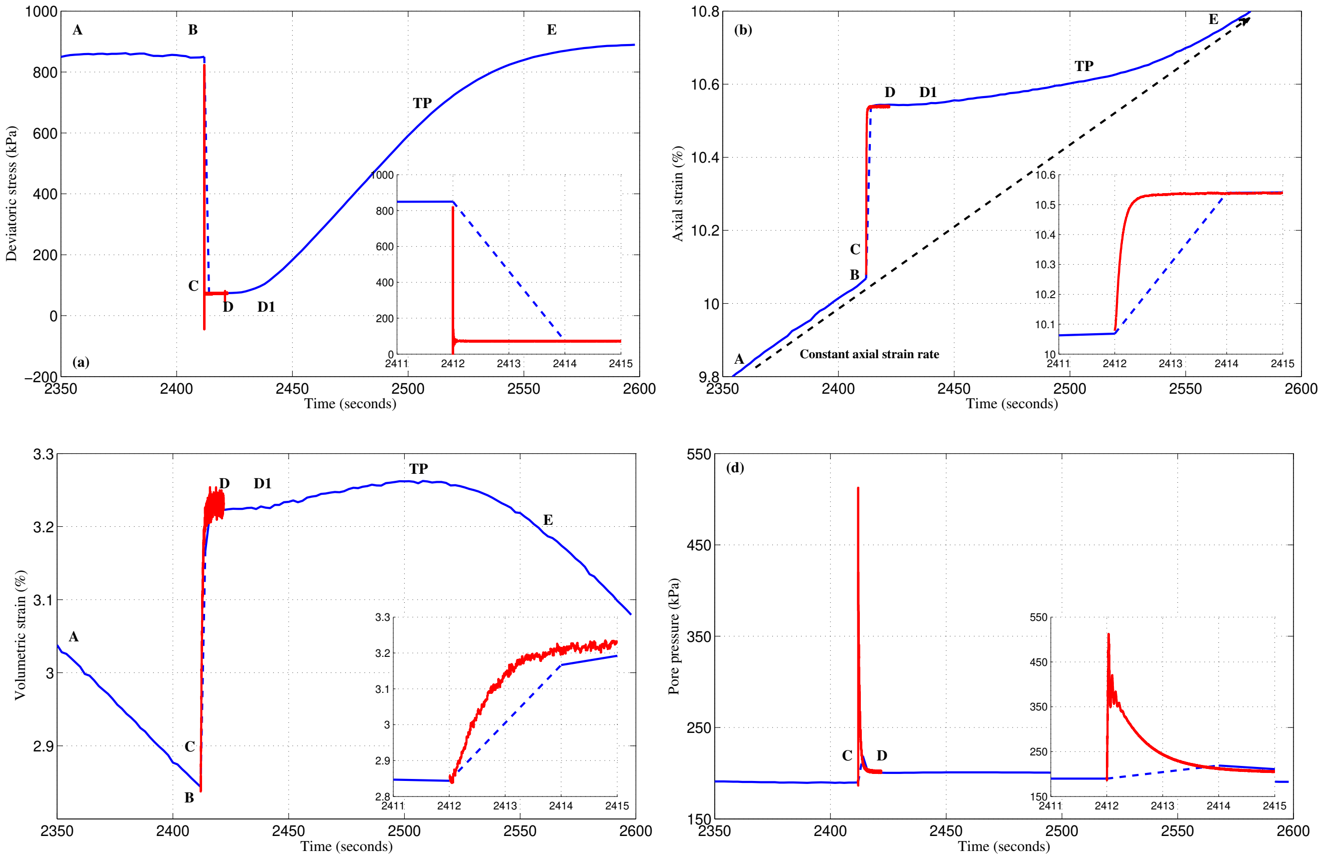

Supplementary Fig. S4. Dynamic behavior on a linear time scale of a typical stick-slip event at the critical stage, SS_7, at a constant confining stress of 400 kPa, with emphasis on the short-lived slip phase (red): (a) Deviatoric stress with a sudden stress drop within 1 ms. Continuous evolution of (b) axial strain behavior, (c) volumetric strain behavior, and (d) pore pressure from one steady state to another. Insets show the temporal behavior within the first 3 s of the slip phase. The dashed blue lines represent the responses presented in the granular literature[1,13–15]. See the text for additional details.

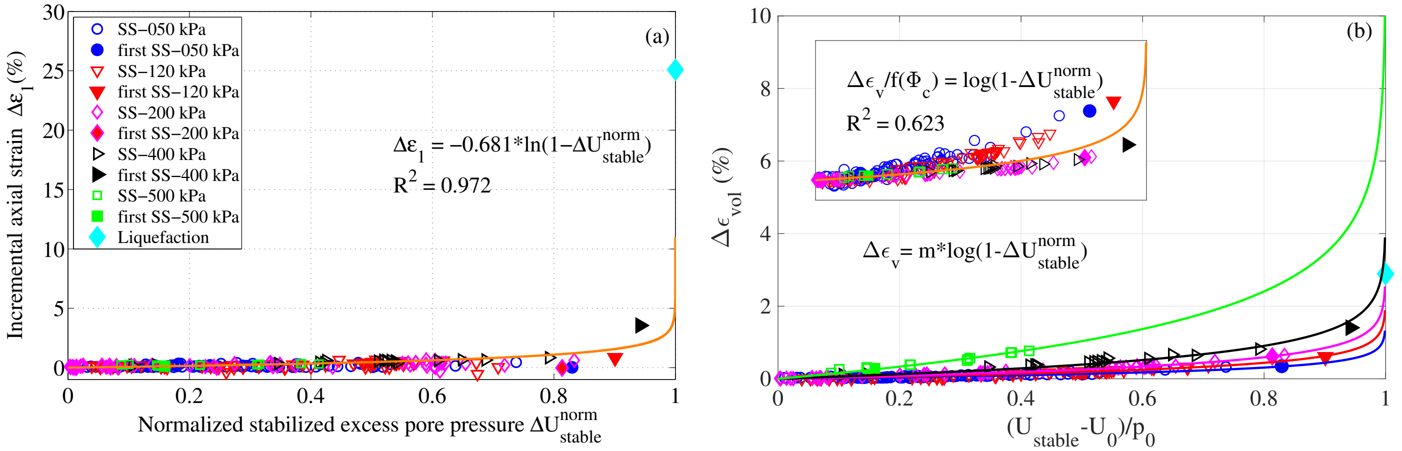

Supplementary Fig. S5. Effects of normalized stabilizing excess pore pressure $(U_{stable} - U_0)/p_0$ and solid fraction Φ_c at constant confining stress from 50 up to 500 kPa on (a) incremental axial strain and (b) incremental volumetric strain with linear scaling relation $f(\Phi_c) = a_f * \Phi_c + b_f$ (a_f = 51.94, b_f = 30.82, R^2 = 0.623). Liquefaction (solid cyan diamond) and first stick-slip motions (solid symbols) are special cases of strong liquefaction potential. Insets show the unique master curve with the scaling parameter as a function of the initial solid fraction at the beginning of the shearing phase Φ_c.

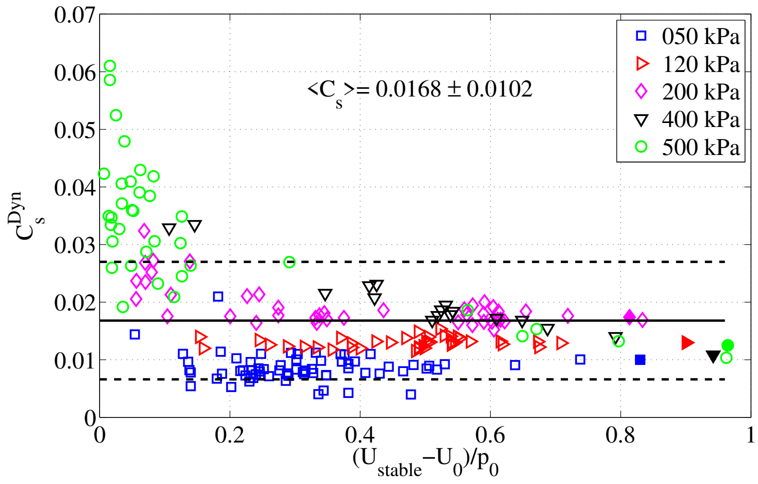

Supplementary Fig. S6. Dynamic consolidation coefficient C_s^{Dyn} of the dynamic slip phase *DE* at different confining stresses from 50 up to 500 kPa. Solid symbols indicate the first stick-slip motions with the highest liquefaction potential $((U_{stable} - U_0)/p_0 \approx 1)$. The horizontal dashed lines give the average values and the 1σ confidence intervals.

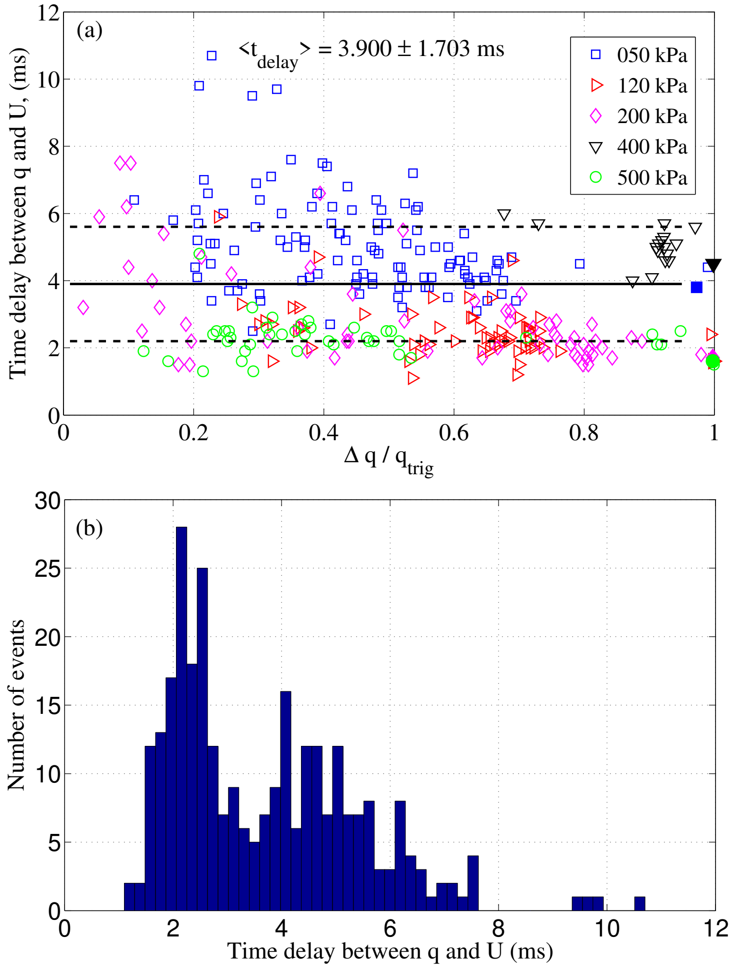

Supplementary Fig. S7. Time delay between deviatoric stress drop and pore pressure generation at different confining stresses from 50 up to 500 kPa. (a) The first stick-slip motions (solid symbols) have the highest normalized deviatoric stress drop ($\Delta q/q_{trig} \approx 1$), decreasing to the isotropic stress level (q ≈ 0). The horizontal dashed lines give the average values and the 1σ confidence intervals. Stick-slip motion under triaxial compression and drained conditions always generates a positive time delay. (b) The corresponding histogram for 264 events.
